# Supplementary figures and images for: Female-germline specific protein Sakura interacts with Otu and is crucial for germline stem cell renewal and differentiation and oogenesis
Source: eLife. 2025 Jul 15;13:RP103828. doi: 10.7554/eLife.103828 (PMC12263153; doi:10.7554/eLife.103828)

Figure 1C

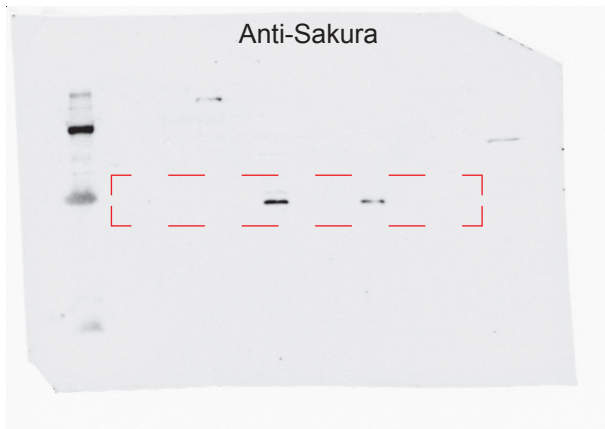

Figure 1D

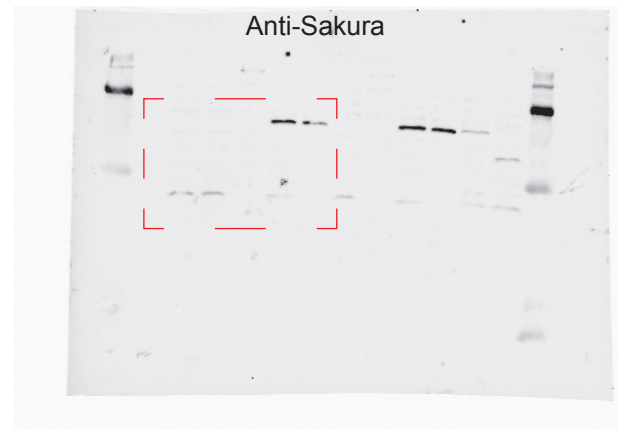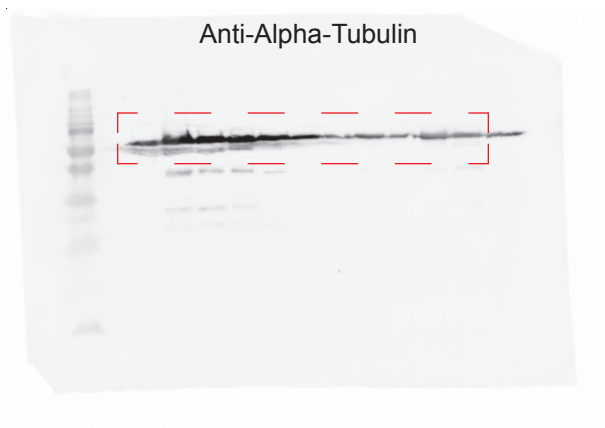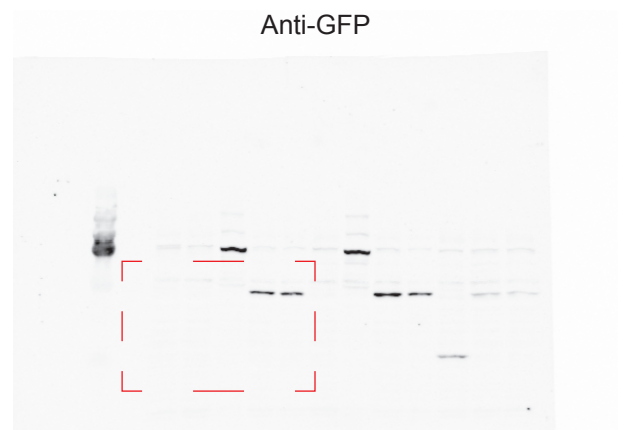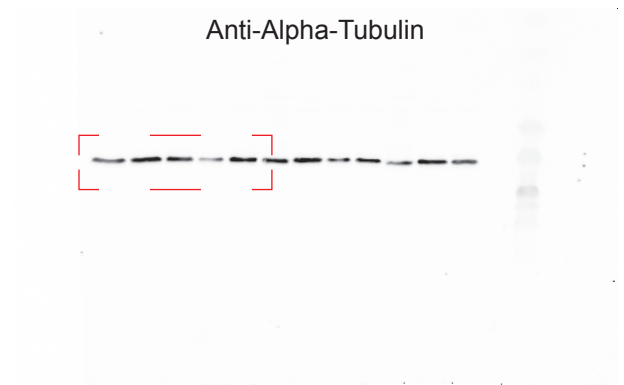

Supplement: Figure 1—source data 1. [file elife-103828-fig1-data1.pdf]

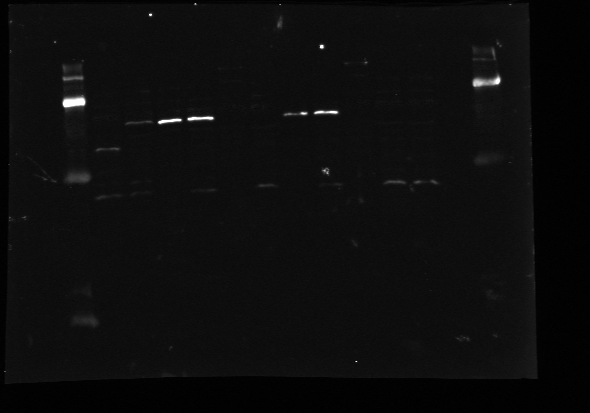

Supplement: Figure 1—source data 2. [file elife-103828-fig1-data2.zip › Figure1_SouceData2/OriginalGelImageFile_Fig1D_800_antiSakura.TIF]

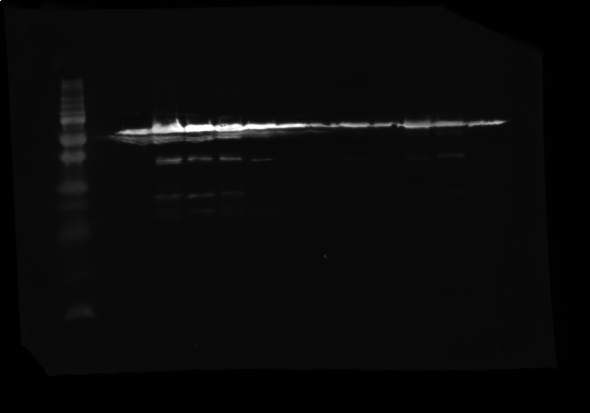

Supplement: Figure 1—source data 2. [file elife-103828-fig1-data2.zip › Figure1_SouceData2/OriginalGelImageFile_Fig1C_700_antiAlphaTubulin.TIF]

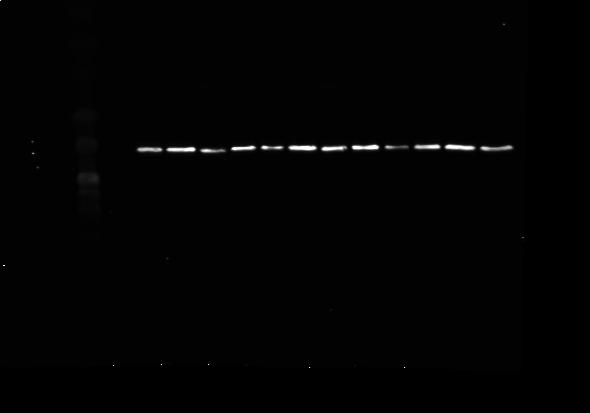

Supplement: Figure 1—source data 2. [file elife-103828-fig1-data2.zip › Figure1_SouceData2/OriginalGelImageFile_Fig1D_700_antiAlphaTubulin.TIF]

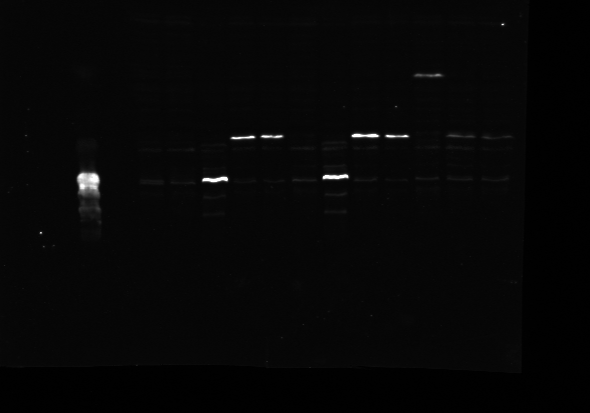

Supplement: Figure 1—source data 2. [file elife-103828-fig1-data2.zip › Figure1_SouceData2/OriginalGelImageFile_Fig1D_800_antiGFP.TIF]

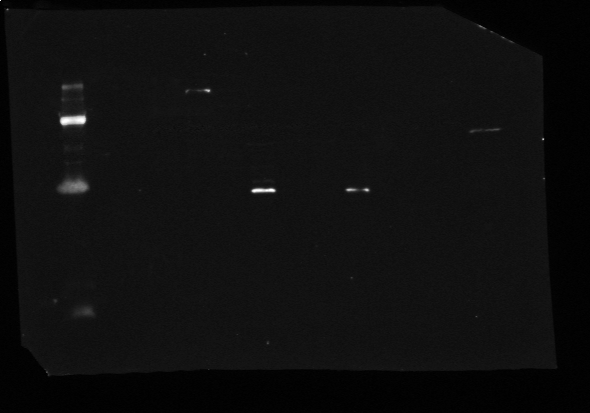

Supplement: Figure 1—source data 2. [file elife-103828-fig1-data2.zip › Figure1_SouceData2/OriginalGelImageFile_Fig1C_800_antiSakura.TIF]

Figure 4-figure supplement 1

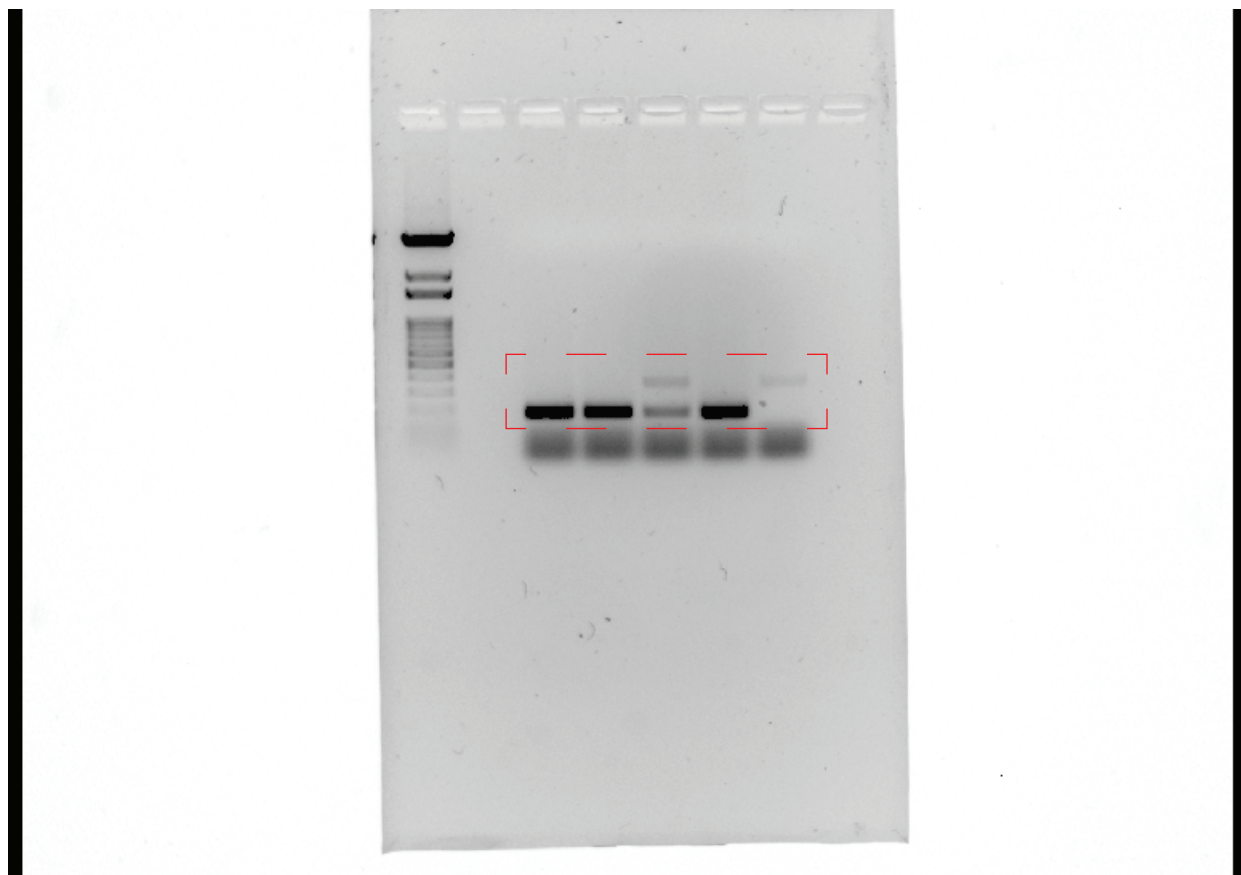

Supplement: Figure 4—figure supplement 1—source data 1. [file elife-103828-fig4-figsupp1-data1.zip › Figure4_SouceData1.pdf]

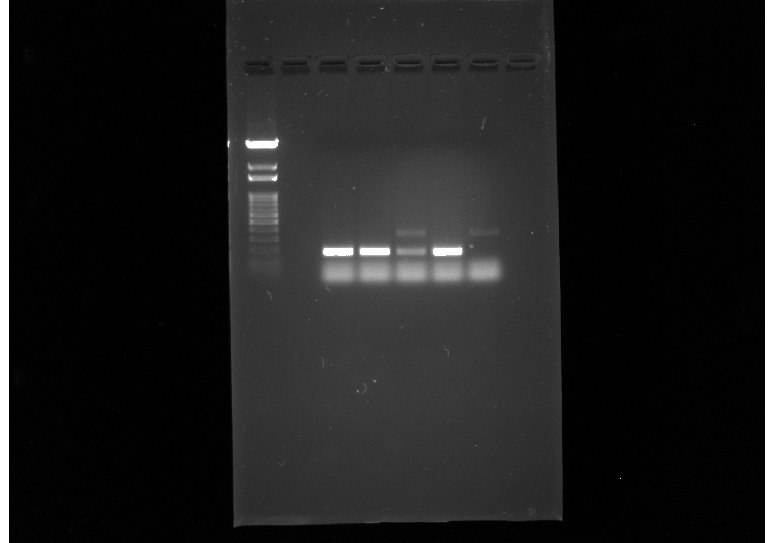

Supplement: Figure 4—figure supplement 1—source data 2. [file elife-103828-fig4-figsupp1-data2.zip › Figure4_SouceData2/OriginalGelImageFile_Fig4sup1.tif]

Figure 5B

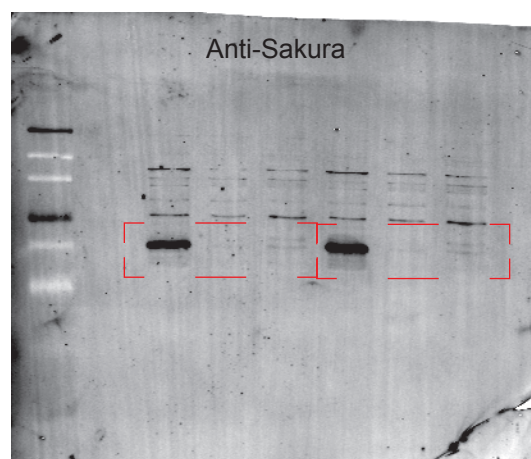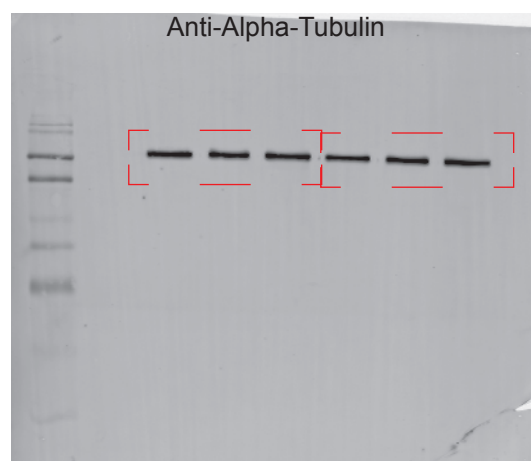

Supplement: Figure 5—source data 1. [file elife-103828-fig5-data1.pdf]

Figure 9D

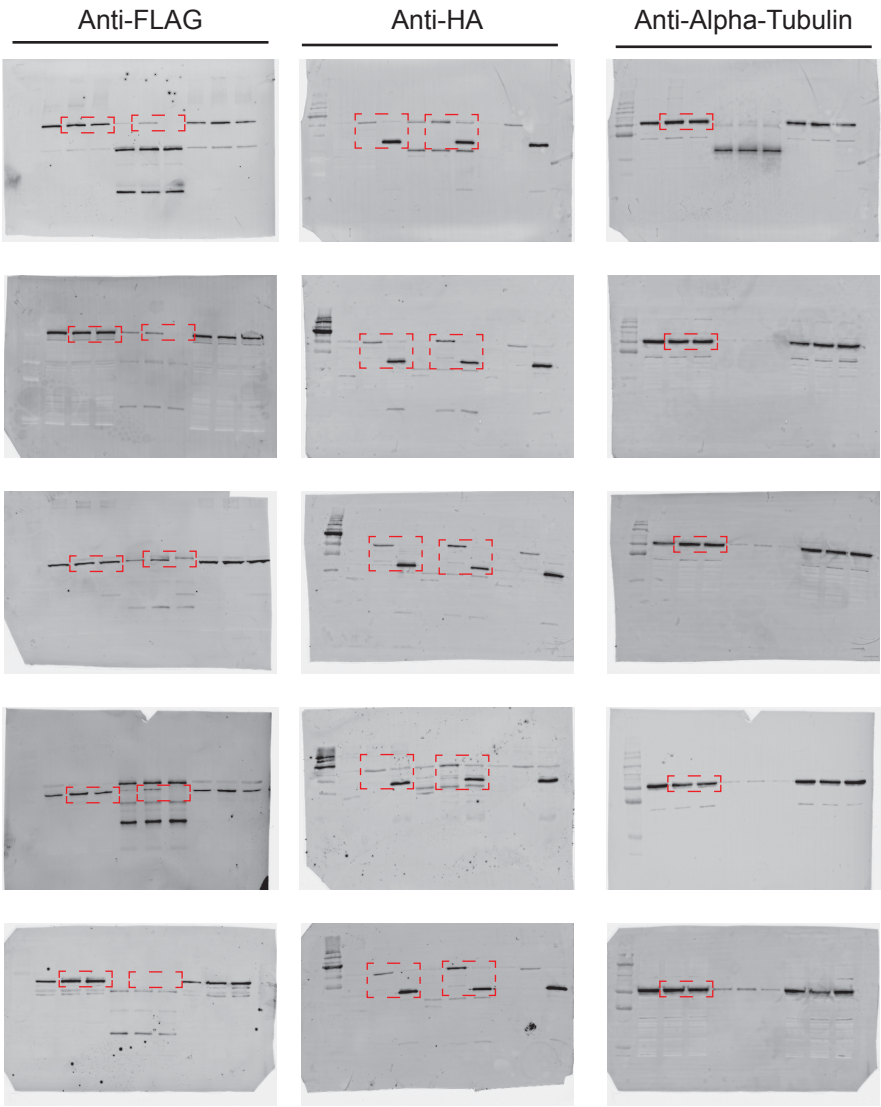

Figure 9E

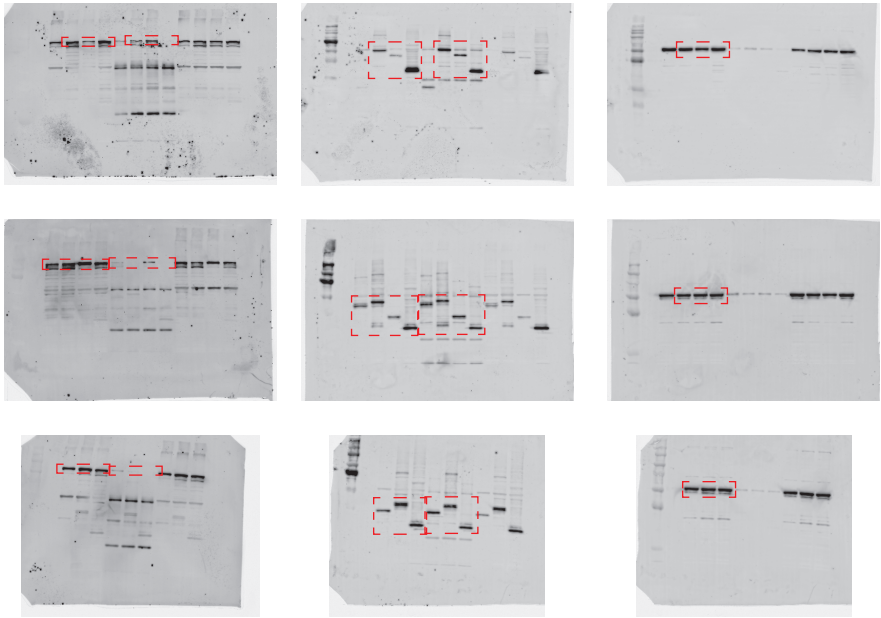

Figure 9F

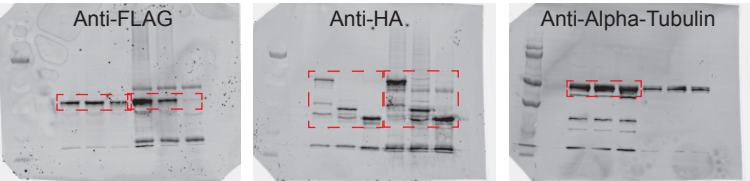

Figure 9-figure supplement 1

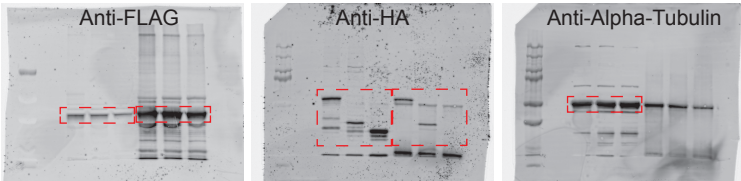

Supplement: Figure 9—source data 1. [file elife-103828-fig9-data1.pdf]
