# Supplementary material for: Female-germline specific protein Sakura interacts with Otu and is crucial for germline stem cell renewal and differentiation and oogenesis
Source: eLife. 2025 Jul 15;13:RP103828. doi: 10.7554/eLife.103828 (PMC12263153; doi:10.7554/eLife.103828)

Figure 8A

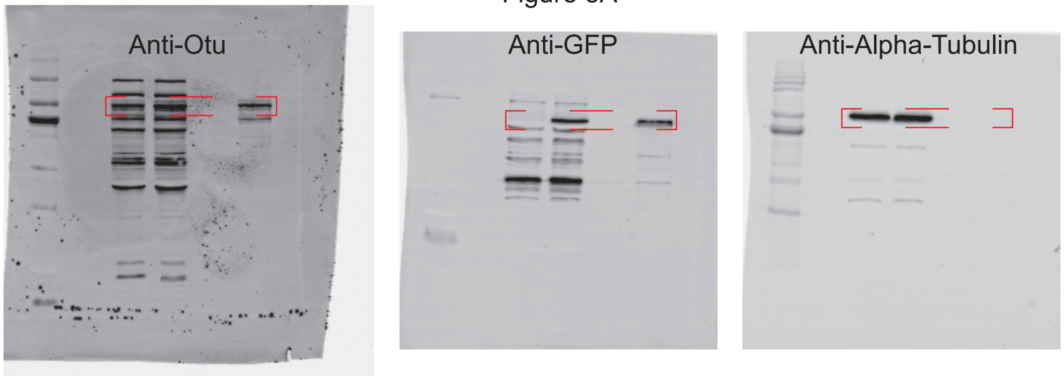

Figure 8B

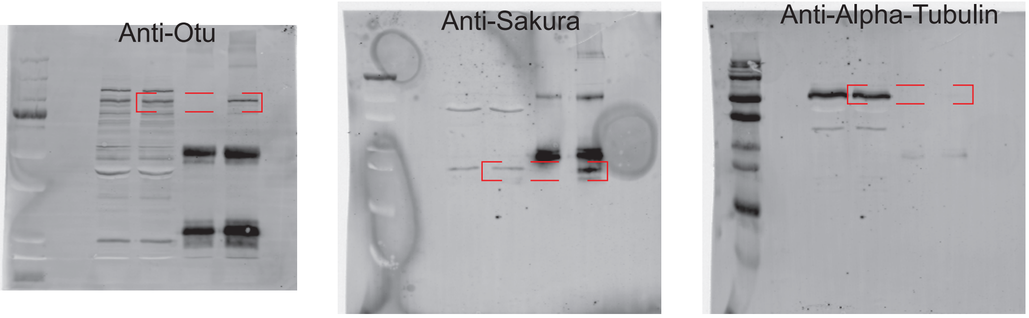

Figure 8C

Anti-FLAG Anti-HA Anti-Alpha-Tubulin

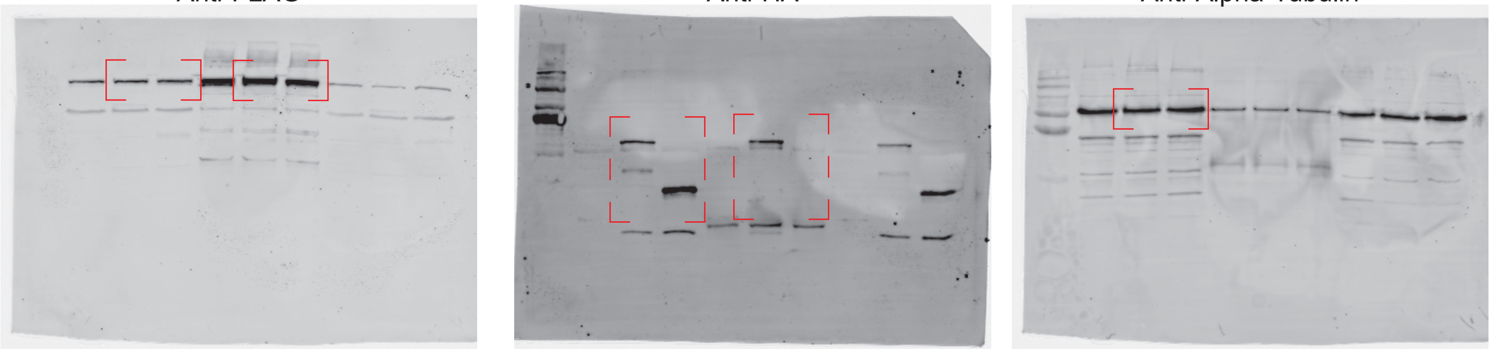

Figure 8-figure supplement 1-A

Anti-Otu Anti-Alpha-Tubulin

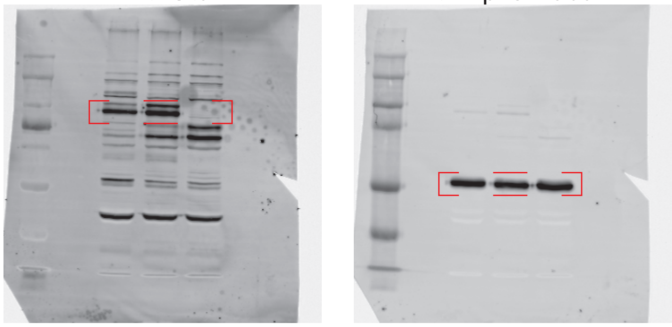

Figure 8-figure supplement 1-B

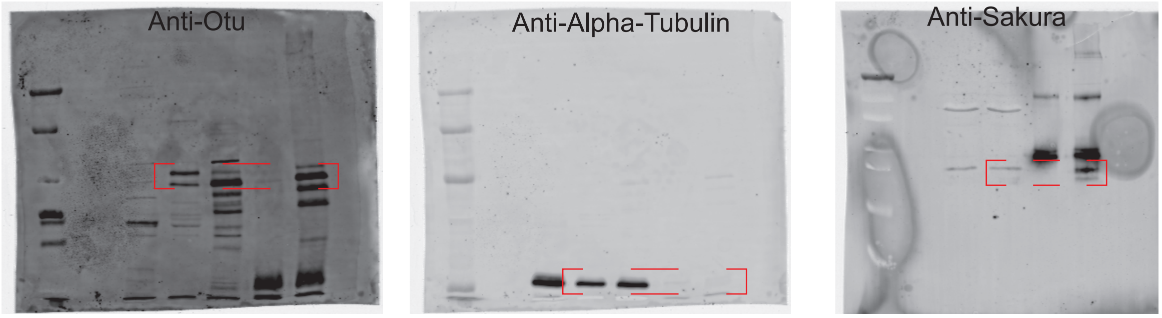

Supplement: Figure 8—source data 1. [file elife-103828-fig8-data1.pdf]
