# Supplementary material for: Female-germline specific protein Sakura interacts with Otu and is crucial for germline stem cell renewal and differentiation and oogenesis
Source: eLife. 2025 Jul 15;13:RP103828. doi: 10.7554/eLife.103828 (PMC12263153; doi:10.7554/eLife.103828)

Figure 10C

Anti-Otu | Anti-Alpha-Tubulin | Anti-Otu | Anti-Alpha-Tubulin

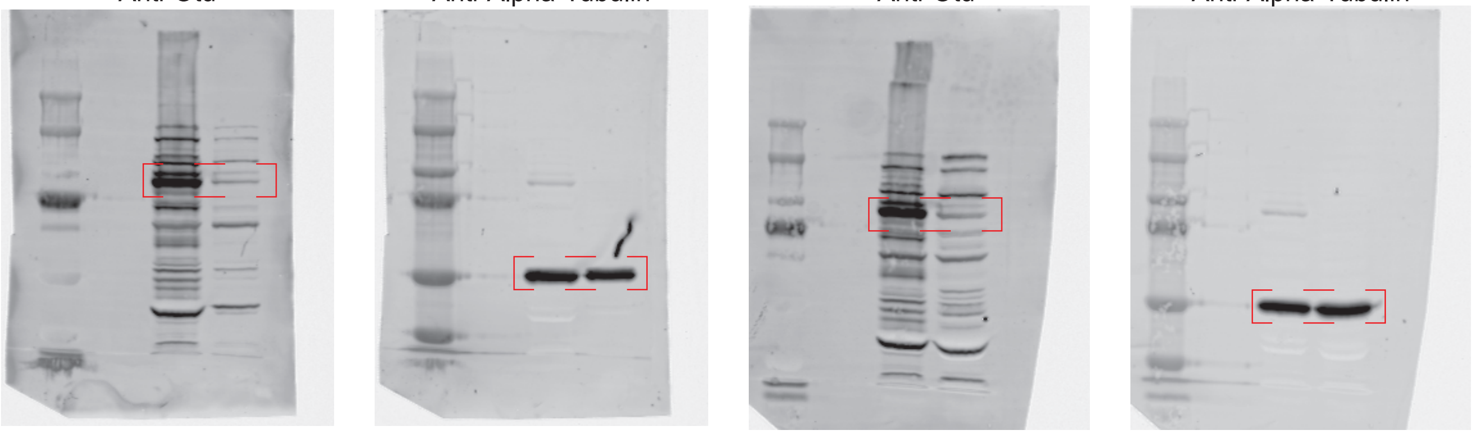

Figure 10-figure supplement 1-A

Anti-Sakura | Anti-Otu | Anti-Alpha-Tubulin

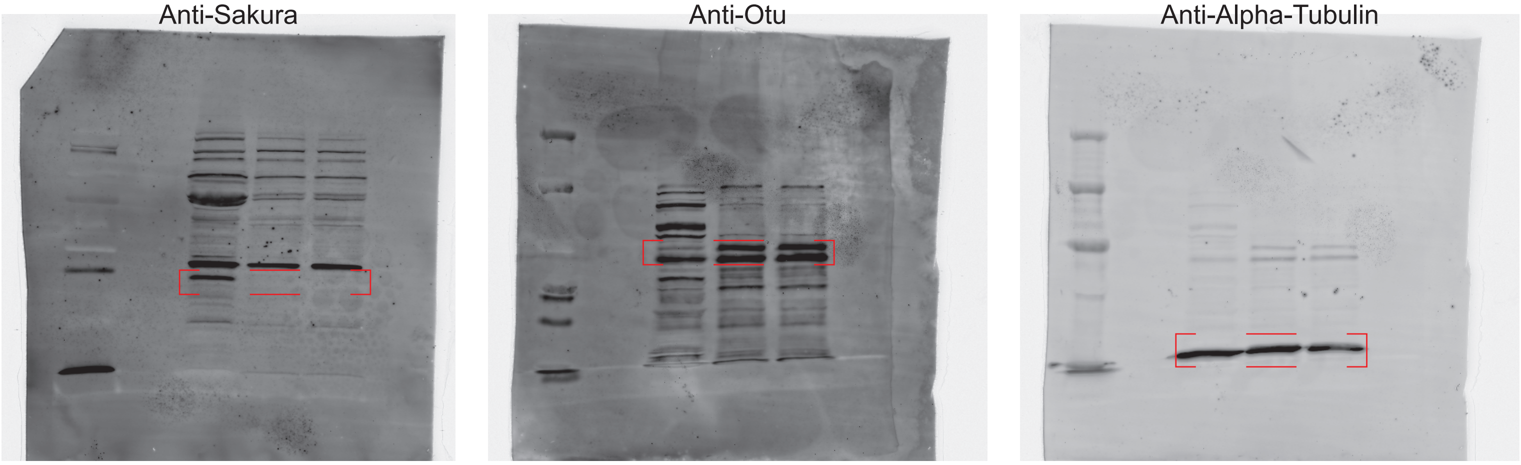

Figure 10-figure supplement 1-B

Anti-Otu | Anti-Sakura | Anti-Alpha-Tubulin

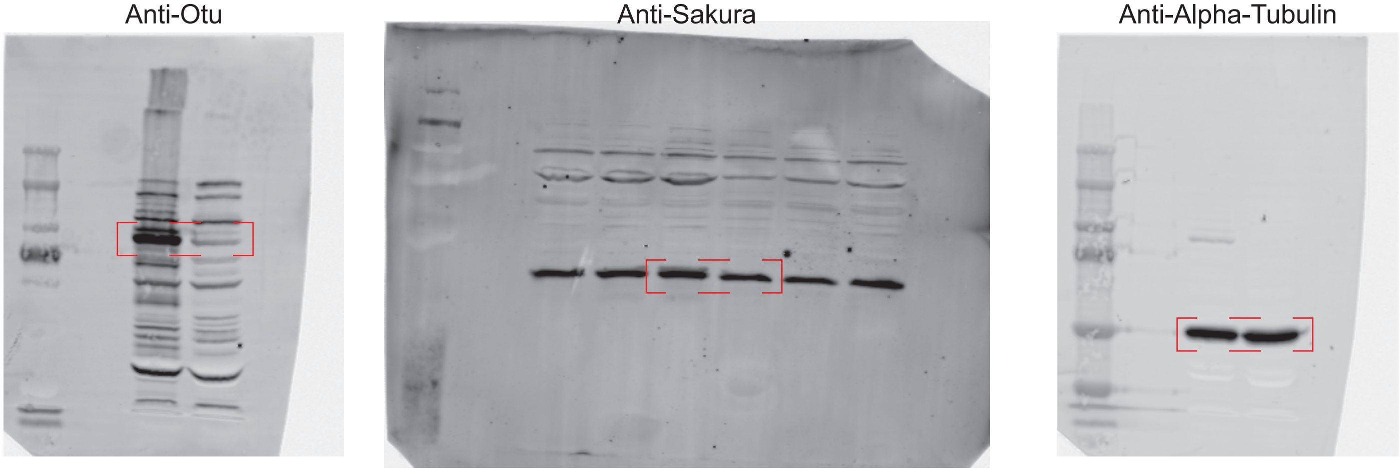

Supplement: Figure 10—source data 1. [file elife-103828-fig10-data1.pdf]
